# The ATP-Mediated Regulation of KaiB-KaiC Interaction in the Cyanobacterial Circadian Clock

**DOI:** 10.1371/journal.pone.0080200

**Published:** 2013-11-11

**Authors:** Risa Mutoh, Atsuhito Nishimura, So Yasui, Kiyoshi Onai, Masahiro Ishiura

**Affiliations:** 1 Center for Gene Research, Nagoya University, Nagoya, Aichi, Japan; 2 Division of Biological Science, Graduate School of Science, Nagoya University, Nagoya, Aichi, Japan; Ohio State University, United States of America

## Abstract

The cyanobacterial circadian clock oscillator is composed of three clock proteins—KaiA, KaiB, and KaiC, and interactions among the three Kai proteins generate clock oscillation *in vitro*. However, the regulation of these interactions remains to be solved. Here, we demonstrated that ATP regulates formation of the KaiB-KaiC complex. In the absence of ATP, KaiC was monomeric (KaiC^1mer^) and formed a complex with KaiB. The addition of ATP plus Mg^2+^ (Mg-ATP), but not that of ATP only, to the KaiB-KaiC^1mer^ complex induced the hexamerization of KaiC and the concomitant release of KaiB from the KaiB-KaiC^1mer^ complex, indicating that Mg-ATP and KaiB compete each other for KaiC. In the presence of ATP and Mg^2+^ (Mg-ATP), KaiC became a homohexameric ATPase (KaiC^6mer^) with bound Mg-ATP and formed a complex with KaiB, but KaiC hexamerized by unhydrolyzable substrates such as ATP and Mg-ATP analogs, did not. A KaiC N-terminal domain protein, but not its C-terminal one, formed a complex with KaiB, indicating that KaiC associates with KaiB *via* its N-terminal domain. A mutant KaiC^6mer^ lacking N-terminal ATPase activity did not form a complex with KaiB whereas a mutant lacking C-terminal ATPase activity did. Thus, the N-terminal domain of KaiC is responsible for formation of the KaiB-KaiC complex, and the hydrolysis of the ATP bound to N-terminal ATPase motifs on KaiC^6mer^ is required for formation of the KaiB-KaiC^6mer^ complex. KaiC^6mer^ that had been hexamerized with ADP plus aluminum fluoride, which are considered to mimic ADP-Pi state, formed a complex with KaiB, suggesting that KaiB is able to associate with KaiC^6mer^ with bound ADP-Pi.

## Introduction

**Figure 1:**
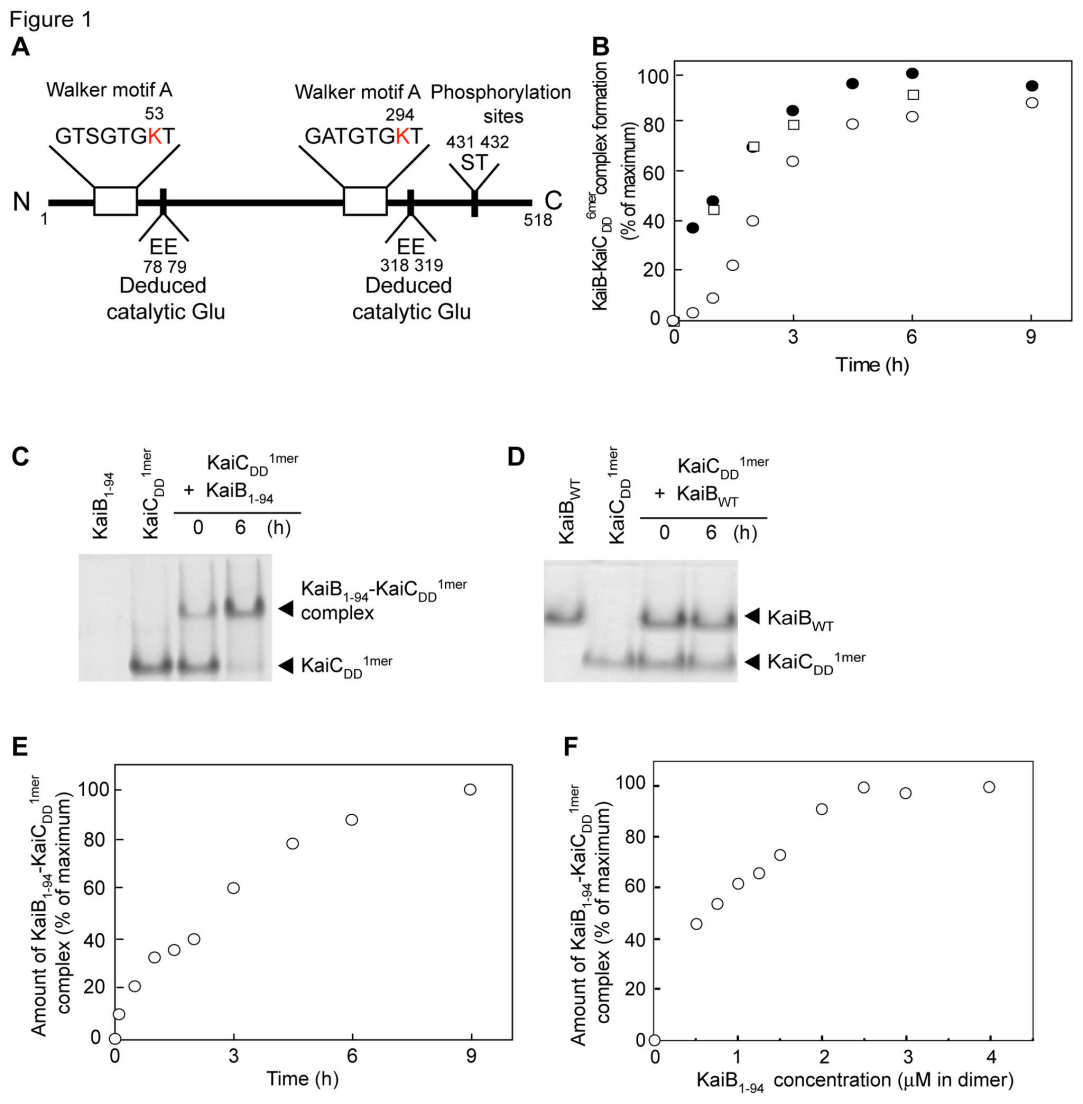
The sequence motifs of KaiC and KaiB-KaiC complex formation. A. A diagram of the sequence motifs of KaiC. B. Time courses of KaiB-KaiC_DD_^6mer^ complex formation. KaiB_WT_ (1 μM) and KaiB_1-94_ (2 μM) were separately incubated with 1 μM KaiC_DD_^6mer^ in reaction buffer containing Mg-ATP at 4 °C or 40 °C for the various periods indicated, and then aliquots of the reaction mixtures were subjected to native PAGE on 10 % gels. After staining the gels with CBB to visualize proteins, we estimated the amounts of KaiBs-KaiC_DD_^6mer^ complex by densitometry. We used the maximum value obtained at a time of 6 h as the maximum value. KaiBs added and temperature conditions: open circles, KaiB_WT_ (40 °C); closed circles, KaiB_1-94_ (40 °C); open squares, KaiB_1-94_ (4 °C). C. A native PAGE gel of the reaction products of KaiC_DD_^1mer^ with KaiB_1-94_. Reaction mixtures containing 15 μM KaiB_1-94_ and 5 μM KaiC_DD_^1mer^ in reaction buffer were incubated at 4 °C for 6 h and then subjected to native PAGE. Proteins were visualized by CBB staining of the gel. Because KaiB_1-94_ has an isoelectric point of 9.7, it moved in the opposite direction and could not detect by native PAGE. D. A native PAGE gel of the reaction products of KaiC_DD_^1mer^ with KaiB_WT_. Reaction mixtures containing 7.5 μM KaiB_WT_ and 5 μM KaiC_DD_^1mer^ in reaction buffer were incubated at 4 °C for 6 h and then subjected to native-PAGE. E. Time course of KaiB_1-94_-KaiC_DD_^1mer^ complex formation. Reaction mixtures containing 5 μM KaiB_1-94_ and 5 μM KaiC_DD_^1mer^ in reaction buffer were incubated at 4 °C for the periods indicated and then subjected to native PAGE. We used the value obtained at a time of 9 h as the maximum value. A typical experimental data is shown. F. KaiB_1-94_ concentration-dependence of the KaiB_1-94_-KaiC_DD_^1mer^ complex formation. Reaction mixtures containing 1 μM KaiC_DD_^1mer^ and various amounts (0.5, 0.75, 1.0, 1.25, 1.5, 2.0, 2.5, 3.0, and 4.0 μM) of KaiB_1-94_ in reaction buffer were incubated at 4 °C for 6 h and then subjected to native PAGE. We used the value obtained at a KaiB_1-94_ concentration of 4 μM as the maximum value. A typical experimental data is shown.

Circadian rhythms—oscillations that regulate metabolic and behavioral activity in approximately 24-h periods—are observed in almost all organisms from prokaryotes to eukaryotes. Cyanobacteria are the simplest organisms to exhibit circadian rhythms [1]. The gene cluster *kaiABC* that is essential for the generation of circadian rhythms has been cloned and analyzed in the cyanobacterium *Synechococcus* sp. strain PCC 7942 (hereafter *Synechococcus*) [2]. The cyanobacterial clock oscillator is composed of only three clock proteins, KaiA, KaiB, and KaiC. Circadian oscillations are generated at the phosphorylation level [3] and ATPase activity [4] of KaiC and the formation of complexes between the proteins [5,6]. KaiC is a homohexameric ATP-binding protein with autokinase activity [7-9], very weak temperature-independent (temperature-compensated) ATPase activity [4,10], and autophosphatase activity [11,12]. The KaiC subunit has a duplicated structure composed of N-terminal and C-terminal domains, and each has a series of ATPase motifs (a Walker's motif A, a Walker's motif B, and a catalytic glutamate (CatE; We named the N-terminal CatE and C-terminal CatE as CatE1 and CatE2, respectively.)) (Figure 1A) [2,13]. KaiC has two phosphorylation sites, Ser431 and Thr432, in its C-terminal domain (Figure 1A). A mutant KaiC with aspartate substitutions at the two phosphorylation sites, KaiC_S431D&T432D_ (KaiC_DD_), is considered to mimic the fully phosphorylated state of KaiC [14], and a mutant KaiC with alanine substitutions at the two phosphorylation sites, KaiC_S431A&T432A_ (KaiC_AA_), is considered to mimic fully unphosphorylated state of KaiC [15]. KaiC_DD_ showed higher affinity with KaiB than KaiC_AA_ [5,15-18]. KaiA is a homodimeric protein [9,19,20] that enhances the phosphorylation level [9,21,22] and ATPase activity [4,10] of KaiC. KaiB is a homotetrameric protein [23-25], and it has a positively charged cleft flanked by two negatively charged ridges [24]. KaiB suppresses the autokinase activity [22,26] and ATPase activity [4] of KaiC. KaiC associates with KaiA [8,9] and KaiB [25,27], and KaiB also associates directly with KaiA [16,28,29]. These interactions are essential for the generation of clock oscillation. On interaction with hexameric KaiC (KaiC^6mer^), tetrameric KaiB (KaiB^4mer^) likely dissociates into two dimeric KaiB (KaiB^2mer^) and forms a complex comprising one molecule of KaiC^6mer^ and two of KaiB^2mer^ [17]. Previously, we have shown that KaiB_1-94_-KaiC_DD_^6mer^ complex (350 ± 20 kDa) consists of two molecules of KaiB_1-94_ (dimeric) and one molecule of KaiC_DD_^6mer^ and also have shown data suggesting that KaiB_WT_-KaiC_DD_^6mer^ complex (366 ± 20 kDa) consists of two molecules of dimerized KaiB_WT_ (KaiB_WT_^2mer^) and one molecule of KaiC_DD_^6mer^ [17]. The histidine kinase SasA, which is the first component of the main output pathway that transduces clock oscillation to genome-wide transcription cycles [30], competes with KaiB to form a complex with KaiC [17], and SasA and KaiC associate through their N-terminal domains [14]. How the KaiB-KaiC interaction is regulated, however, remains unknown.

 Here, we investigated the interaction of KaiC with KaiB *in vitro* and found that KaiC associates with KaiB *via* its N-terminal domain and that ATP regulates the KaiB-KaiC interaction. We propose a model for ATP regulation of KaiB-KaiC interaction.

## Materials and Methods

### Preparation of Kai protein

 We produced recombinant Kai proteins derived from the thermophylic cyanobacterium *Thermocynechococcus elongatus* BP-1 using *Escherichia coli* as a host, as described previously, with modifications [13]. The plasmids expressing KaiA whose subunit consists of 283 amino acid residues, wild-type KaiB (KaiB_WT_; p*Te*kaiB_WT_), whose subunit consists of 108 amino acid residues, a mutant KaiB with a C-terminal deletion of residues 95 to 108 (KaiB_1-94_; p*Te*kaiB_1-94_), wild-type KaiC (KaiC_WT_; p*Te*kaiC_WT_), whose subunit consists of 518 amino acid residues, and mutant KaiCs— a mutant KaiC with aspartate substitutions at the two phosphorylation sites that is considered to mimic the fully phosphorylated state of KaiC, KaiC_S431D&T432D_ (KaiC_DD;_ p*Te*kaiC_DD_), a mutant KaiC with alanine substitutions at the two phosphorylation sites that is considered to mimic fully unphosphorylated state of KaiC, KaiC_S431A&T432A_ (KaiC_AA_; p*Te*kaiC_AA_), a mutant KaiC with glutamine substitutions at the two deduced catalytic glutamate residues of the C-terminal ATPase motifs that lacks the C-terminal ATPase activity of KaiC, KaiC_CatE2_- (p*Te*kaiC_CatE2_-), KaiC_CatE2_-_/S431A&S432A_ (KaiC_CatE2_-_/AA_; p*Te*kaiC_CatE2_-_/AA_), an N-terminal domain mutant protein of residues 1 to 268 (KaiC_N_; p*Te*kaiC_N_), and a C-terminal domain mutant protein of residues 269 to 518 with a mutation with aspartate substitutions at the two KaiC phosphorylation sites (KaiC_C/DD_; p*Te*kaiC_C/DD_)—have been described previously [8,28]. We constructed plasmids for the production of KaiC_K53H/ DD_ (p*Te*kaiC_K53H/DD_), which is deficient in the N-terminal ATP binding site (N-terminal ATPase motifs) and carries aspartate substitutions at the two phosphorylation sites on the C-terminal domain (DD mutation), and KaiC_CatE1_-_/DD_ (p*Te*kaiC_CatE1_-_/DD_) , which is deficient in the N-terminal ATPase and carries the DD mutation, by replacing a 1.25-kb *Bam*HI-*Eco*RI fragment carrying the 3'-region of each *kaiC* gene in p*Te*kaiC_K53H_ and p*Te*kaiC_CatE1_-, respectively, with that of p*Te*kaiC_DD_. We constructed a plasmid for KaiC_K294H/DD_ (p*Te*kaiC_K294H/DD_), which is deficient in the N-terminal ATP binding site and carries the DD mutation as descried previously [13]. We purified KaiA, KaiB, and KaiC as described previously [28] and stored them at -85 °C until used. KaiA is a dimer [9,19,20], KaiB_WT_ tetramer [23-25], and KaiB_1-94_ dimer [17]. Thus unless otherwise stated, we expressed the concentrations of KaiA, KaiB_WT_, KaiB_1-94_, KaiC^1mer^, and KaiC^6mer^ as those of a dimer, tetramer, dimer, monomer, and hexamer, respectively.

### Preparation of KaiC^6mer^

 To allow the hexamerization of KaiC, we incubated KaiC^1mer^ with 1 mM ATP or 1 mM ATP analogs, 5’-adenylylimidodiphosphate (AMPPNP) and adenosine 5'-O-(3-thio) triphosphate (ATPγS), in the presence of 5 mM MgCl_2_ in 20 mM Tris-HCl buffer (pH 7.5) containing 150 mM NaCl at 20 °C for 20 min. We then subjected the reaction mixtures to gel filtration chromatography on a Superdex 200/HR 10/30 column (GE Healthcare) equilibrated with 20 mM HEPES-NaOH buffer (pH 7.5) containing 150 mM NaCl (reaction buffer), 0.1 mM ATP (or ATP analogs) and 5 mM MgCl_2_ at 4 °C, and we collected fractions containing KaiC^6mer^. We similarly prepared KaiC^6mer^ in the absence of MgCl_2_. Unless otherwise stated, KaiC^6mer^ means KaiC hexamerized with 1 mM ATP plus 5 mM MgCl_2_ (Mg-ATP). The phosphorylation level of the KaiC_WT_ preparations used here was about 30 %.

### Assay for the complex formation of monomeric KaiC (KaiC^1mer^) with KaiBs by native polyacrylamide gel electrophoresis (native PAGE)

 We incubated reaction mixtures containing 15 μM KaiB_1-94_ or 7.5 μM KaiB_WT_ and 5 μM KaiC^1mer^ or 2.5 μM KaiC^6mer^ at 4 °C in reaction buffer and subjected 20-μl aliquots to native PAGE on 10 % gels (acrylamide: bisacrylamide = 37.5: 1) and stained the gels with Coomassie Brilliant Blue (CBB). We estimated the amount of KaiB-KaiC^1mer^ complex by densitometry using a Lane Analyzer (ATTO, Tokyo, Japan) and a CS Analyzer (ATTO).

### Assay for formation of the KaiB_1-94_-KaiC^6mer^ complex by native-PAGE and 2-dimensional sodium dodecyl sulfate (SDS)-PAGE

 We incubated reaction mixtures containing 10 μM KaiB_1-94_ and 6 μM KaiC^6mer^ at 4, 25, or 40 °C in reaction buffer and subjected aliquots to native PAGE. We confirmed the presence of KaiB_1-94_ and KaiC in the complex bands by native PAGE followed by SDS-PAGE on 18 % gels, as described previously [8].

### Assay for formation of the KaiB_1-94_-KaiC^1mer^ complex and KaiB_1-94_-KaiC^6mer^ complex by gel filtration chromatography

 To assay for formation of the KaiB_1-94_-KaiC^1mer^ complex and the KaiB_1-94_-KaiC^6mer^ complex, we incubated reaction mixtures containing 36 μM KaiB_1-94_ and 12 μM KaiC^1mer^ in reaction buffer and those containing 7.5 μM KaiB_1-94_ and 2.5 μM KaiC^6mer^ (KaiC_WT_ or mutant KaiCs) in the buffer with (for KaiC^6mer^) or without (for KaiC^1mer^) Mg-ATP at 4 °C for 6 h. We then analyzed the mixtures by gel filtration chromatography on a Superdex 200/HR 10/30 column equilibrated with reaction buffer with (for KaiC^6mer^) or without (for KaiC^1mer^) 0.1 mM ATP and 5 mM MgCl_2_ at 4 °C. We subjected all peak fractions to SDS-PAGE and visualized by staining with CBB.

### Immunoblot analysis

 Because we could not easily detect the KaiB_1-94_ contained in a putative complex between KaiB_1-94_-KaiC_N_^6mer^ by CBB staining of SDS-PAGE gels, we subjected the gels to immunoblotting. We incubated reaction mixtures containing KaiB_1-94_ and/or KaiC_N_^6mer^ at 4 °C for 6 h as described above, and then subjected them to gel filtration chromatography on a Superdex 200/HR 10/30 column equilibrated with reaction buffer containing 0.5 mM ATP and 5 mM MgCl_2_ at 4 °C. We subjected fractions containing a putative KaiB_1-94_-KaiC_N_^6mer^ complex to SDS-PAGE, blotted the proteins onto Immobilon-P Transfer Membrane (Millipore), and visualized them using the ECL Western Blotting Analysis System (GE Healthcare) with a rabbit anti-KaiB antiserum (diluted to 1/2000) as a primary antibody and a donkey anti-rabbit Ig antibody (GE Healthcare) as a secondary antibody, as described previously [8].

### Assay for the time course of formation of the KaiB_1-94_-KaiC_DD_^1mer^ complex

 We incubated KaiC_DD_^1mer^ (5 μM) with 5 μM KaiB_1-94_ in reaction buffer at 4 °C for various periods and subjected aliquots of the reaction mixtures to native PAGE on 10 % gels, staining the gels with CBB as described above. We estimated the amount of KaiB_1-94_-KaiC_DD_^1mer^ complex from the intensity of the band by densitometry as described above. 

### KaiB_1-94_-concentration dependence of KaiB-KaiC^1mer^ complex formation

 We incubated 1 μM KaiC_DD_^1mer^ with various amounts of KaiB_1-94_ (0.5, 0.75, 1.0, 1.25, 1.5, 2.0, 2.5, 3.0, and 4.0 μM) in reaction buffer at 4 °C for 6 h. Other conditions were the same as described above. 

### Assay for Mg-ATP-induced dissociation of the KaiB_1-94_-KaiC^1mer^ complex

 We assayed the ATP-induced hexamerization of KaiC_DD_^1mer^ in the KaiB_1-94_-KaiC^1mer^ complex and the concomitant release of KaiB_1-94_. To obtain the KaiB_1-94_-KaiC_DD_^1mer^ complex, we incubated reaction mixtures containing 24 μM KaiB_1-94_ and 24 μM KaiC_DD_^1mer^ in reaction buffer at 4 °C for 16 h and then subjected the mixtures to gel filtration chromatography on a Superdex 75/HR 10/30 column (GE Healthcare) equilibrated with reaction buffer at 4 °C. We added 1 mM ATP with or without 5 mM MgCl_2_ to the obtained KaiB_1-94_-KaiC_DD_^1mer^ complex and then incubated the reaction mixtures in reaction buffer at 4 °C for 6 h. Then, we subjected the reaction mixtures to gel filtration chromatography on a Superdex 200/HR 10/30 column equilibrated with reaction buffer at 4 °C. We analyzed all peak fractions by SDS-PAGE on 18 % gels, and then stained the gels with CBB.

### Preparation of KaiC^6mer^ formed with ADP and aluminum fluoride (KaiC^6mer^ (ADP-AlF_X_)) and assay for the KaiB_1-94_-KaiC^6mer^ (ADP-AlF_X_) complex

 We incubated KaiC^1mer^ (20 μM) in reaction buffer containing 6 mM ADP, 30 mM MgCl_2_, 2.5 mM NaF, and 2.5 mM AlCl_3_ at 25 °C for 2 h to form KaiC^6mer^ (ADP-AlF_X_) [31]. We then mixed the reacion mixtures with an equal volume of 0 or 60 μM KaiB_1-94_ in the same buffer and incubated them further at 25 °C for 6 h. We subjected the reaction mixtures to gel filtration chromatography on a Superdex 200/HR 10/30 column equilibrated with reaction buffer at 4 °C. We subjected peak fractions to SDS-PAGE and visualized the proteins by staining with CBB. 

### Assay for the KaiA-enhanced autophosphorylation of KaiC^6mer^

 We incubated reaction mixtures containing 0.5 μM KaiC^6mer^, 0.5 μM KaiA, and Mg-ATP in reaction buffer at 40 °C for various periods. We then subjected aliquots of the reaction mixtures to SDS-PAGE on 12.5 % gels (acrylamide: bisacrylamide = 144: 1), and stained the gels with CBB. We estimated the amount of the protein from the intensities of bands by densitometry as described above.

### Assay for ATPase activity

 We measured the ATPase activity of KaiC^6mer^ using BIOMOL GREEN (Enzo Life Science International, Inc., Farmingdale, New York, USA) according to the supplier's instruction manual. We incubated KaiC^6mer^ (1 μM) in reaction buffer containing Mg-ATP at 4 °C for 6 h, and then incubated 50-μl aliquots of the reaction mixtures with 50 μl BIOMOL GREEN at 25 °C for 20 min. We measured the absorbance of the samples at 620 nm (*A*_*620*_) using an ARVO *X4* plate reader (PerkinElmer Inc., Waltham, MA, USA), calculated the amount of Pi released from ATP from triplicate experiments, and expressed the ATPase activity as mol Pi released per mol KaiC^6mer^ per h at 4 °C. We showed the values after subtracting the buffer background (0.024 ± 0.03 ATP molecules/h). 

## Results

### Association of KaiC^1mer^ with KaiB_1-94_

 At 40 °C, KaiB_WT_ formed a complex with KaiC_DD_^6mer^ (Figure 1B) but the complex formation took more than 9 h to reach a plateau (Figure 1B). KaiB_1-94_-KaiC_DD_^6mer^ complex formation, on the other hand, reached a plateau within 6 h with a time (*t*_*1/2*_) of 1.2 h where the half maximal KaiB_1-94_-KaiC^6mer^ complex formation occurred, and even at 4 °C showed no lag (Figure 1B). Thus, to analyze the formation of the KaiB-KaiC complex in detail, we used KaiB_1-94_ and KaiC_DD_ at 4 °C.

 First, we examined KaiC_DD_^1mer^ complex formation with KaiB_1-94_ and KaiB_WT_. When we incubated 5 μM KaiC_DD_^1mer^ with 15 μM KaiB_1-94_ or 7.5 μM KaiB_WT_ at 4 °C for 6 h, KaiC_DD_^1mer^ formed a complex with KaiB_1-94_ (Figure 1C and Table S1) as KaiC_DD_^6mer^ (Figure 1B), whereas it showed only weak complex formation with KaiB_WT_ (Figure 1D). The complex formation occurred without delay and reached a plateau at 6 h (Figure 1E). The time (*t*_*1/2*_) where the half maximal KaiB_1-94_-KaiC^1mer^ complex formation occurred at 4 °C was about 2.5 h (Figure 1E). The concentration of KaiB_1-94_ where the half maximal KaiB_1-94_-KaiC^1mer^ complex formation occurred was about 0.7 μM (Figure 1F). This value falls within the concentration range at which clock oscillations occur in the *in vitro* KaiABC clock system [5].

### KaiB-interacting domain of KaiC

**Figure 2:**
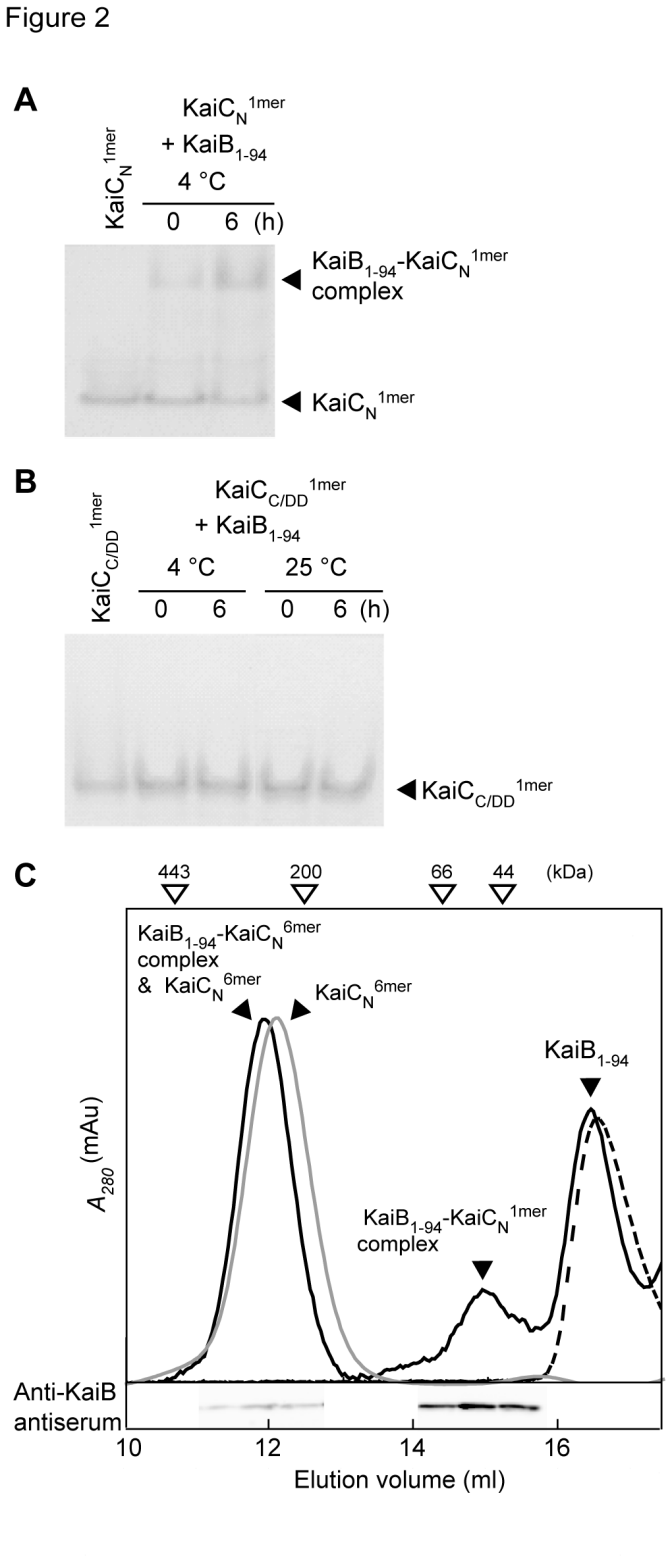
Native PAGE gels, gel filtration chromatography elution profiles, and the immunoblots of the KaiB_1-94_-KaiC complexes. A, B. Native PAGE gels. Reaction mixtures containing 15 μM KaiB_1-94_ and 5 μM KaiC_N_^1mer^ (A) or KaiC_C/DD_^1mer^ (B) were incubated at 4 °C for the periods indicated (hereafter, unless otherwise stated, 0 h shows data for samples taken at the onset of incubation) and subjected to native PAGE. Other conditions were the same as for Figure 1C legend. C. Gel filtration chromatography elution profiles and an immunoblot. Reaction mixtures containing 7.5 μM KaiB_1-94_, 2.5 μM KaiC_N_^6mer^, and 1 mM ATP plus 5 mM MgCl_2_ (Mg-ATP) were incubated at 4 °C for 6 h and then subjected to gel filtration chromatography on a Superdex 200/HR 10/30 column equilibrated with reaction buffer containing 0.5 mM ATP and 5 mM MgCl_2_ at 4 °C. We also separately analyzed KaiB_1-94_ and KaiC_N_^6mer^ by gel filtration chromatography as controls. The peak fraction samples were subjected to SDS-PAGE, blotted to PVDF membranes, and reacted with an anti-KaiB antiserum. Other conditions were the same as for Figure 1C legend. Black solid line, KaiB_1-94_ + KaiC_N_^6mer^; gray solid line, KaiC_N_^6mer^; black broken line, KaiB_1-94_.

 The KaiC subunit is composed of an N-terminal domain (KaiC_N_) and a C-terminal domain (KaiC_C_) [32]. When we incubated 5 μM of KaiC_DD_ monomeric domain protein (KaiC_N_^1mer^ or KaiC_C/DD_^1mer^) with 30 μM KaiB_1-94_ at 4 °C for 6 h, only KaiC_N_^1mer^ formed a complex with KaiB_1-94_ (Figures 2A and B, and Table S1). Even when the reaction mixtures were incubated at 25 °C, we did not detect any KaiB_1-94_-KaiC_C/DD_^1mer^ complex (Figure 2B). Thus, the KaiB-interacting site of KaiC was located on the N-terminal domain of KaiC. 

 When we incubated 2.5 μM KaiC_N_^6mer^ with 7.5 μM KaiB_1-94_ in the presence of Mg-ATP, KaiC_N_^6mer^ showed a weak association with KaiB_1-94_ that was detected by gel filtration chromatography but not by Native PAGE (Figure 2C and Table S2). Unexpectedly, most KaiC_N_^6mer^ became monomeric when it formed a complex with KaiB_1-94_ (Figure 2C). KaiC_C/DD_^6mer^, on the other hand, did not form a complex with KaiB_1-94_ (Table S2). KaiC^6mer^, therefore, also associates with KaiB_1-94_ *via* its N-terminal domain.

### Mg-ATP-induced dissociation of the KaiB_1-94_-KaiC^1mer^ complex

**Figure 3:**
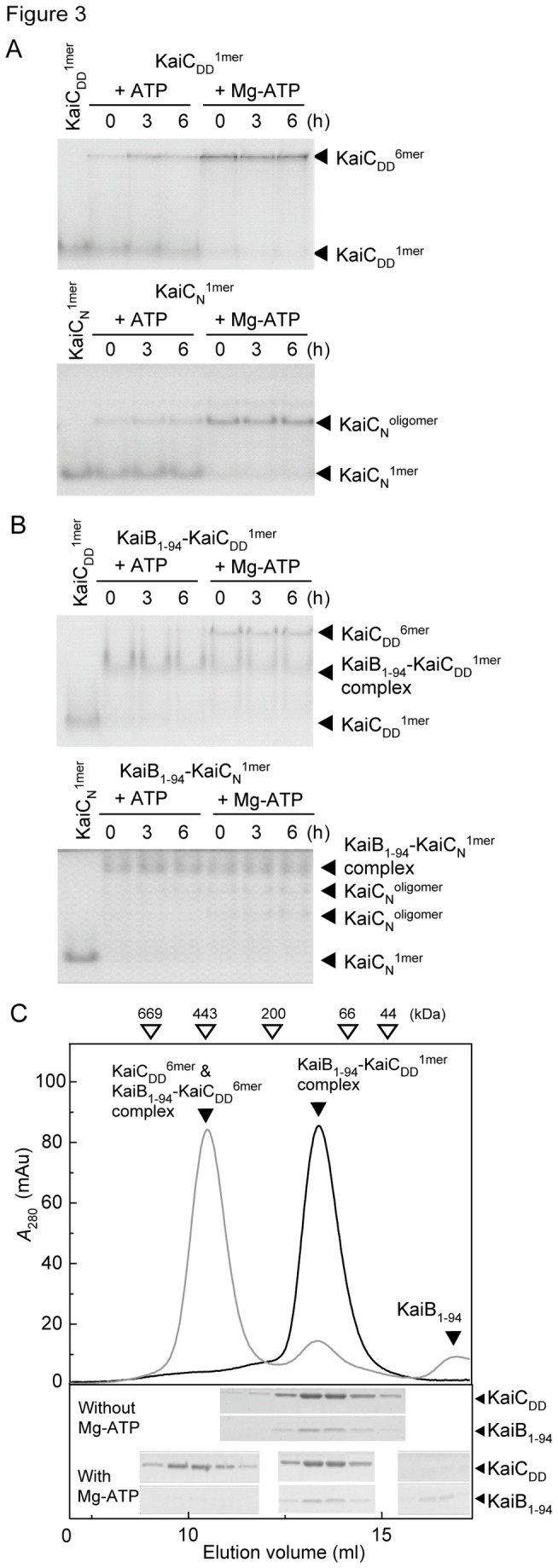
Mg-ATP-induced dissociation of the KaiB_1-94_-KaiC^1mer^ complex. A. Native PAGE gels showing the ATP-induced hexamerization of KaiC_DD_^1mer^ and KaiC_N_^1mer^ in the presence or absence of MgCl_2_. KaiCs^1mer^ (5 μM) were hexamerized by incubation with 1 mM ATP with (Mg-ATP) or without (ATP) 5 mM MgCl_2_ at 4 °C for the periods indicated. Other conditions were the same as described for Figure 1C legend. B. Native PAGE gels of the KaiB_1-94_-KaiC^1mer^ complex after incubation with ATP or Mg-ATP. Reaction mixtures containing 15 μM KaiB_1-94_ and 5 μM KaiC_DD_^1mer^ in reaction buffer were incubated at 4 °C for 16 h to allow the formation of the KaiB_1-94_-KaiC^1mer^ complex. After addition of 1 mM ATP with (Mg-ATP) or without (ATP) 5 mM MgCl_2_ to the complex, the reaction mixtures were further incubated in reaction buffer at 4 °C for 6 h. Other conditions were the same as described for Figure 1C. C. Gel filtration chromatography elution profiles of the KaiB_1-94_-KaiC_DD_^1mer^ complex incubated with or without Mg-ATP. Reaction mixtures containing 24 μM KaiB_1-94_ and 24 μM KaiC_DD_^1mer^ in reaction buffer were incubated at 4 °C for 16 h and then subjected to gel filtration chromatography on a Superdex 75/HR 10/30 column equilibrated with reaction buffer at 4 °C, and KaiB_1-94_-KaiC_DD_^1mer^ complex fractions were collected. With (gray) or without (black) addition of Mg-ATP to the complex, the reaction mixtures were further incubated in reaction buffer at 4 °C for 6 h and then subjected to gel filtration chromatography on a Superdex 200/HR 10/30 column equilibrated with reaction buffer. The peak fractions were subjected to SDS-PAGE. Other conditions were the same as described for Figure 1C. Left and right gels, the 1st and 3rd peak fractions of the reaction products with addition of Mg-ATP, respectively; middle gels, the peak fraction products without addition of Mg-ATP corresponding to the 2nd peak fraction of the reaction products with addition of Mg-ATP.

 Addition of ATP as well as Mg-ATP was able to hexamerize KaiC_DD_^1mer^ and KaiC_N_^1mer^ (Figure 3A) as described previously [33]. When the KaiB_1-94_-KaiC_DD_^1mer^ complex was incubated in the presence of 1 mM ATP or 1 mM ATP plus 5 mM MgCl_2_ (Mg-ATP) at 4 °C for 6 h, Mg-ATP, but not ATP, induced the hexamerization of KaiC_DD_ (Figure 3B) and the concomitant release of KaiB_1-94_ (Figures 3B and 3C). We also detected a small amount of the KaiB_1-94_-KaiC_DD_^6mer^ complex (Figure 3C), which was likely formed from the KaiC_DD_^6mer^ hexamerized by Mg-ATP, and the KaiB_1-94_ released from the KaiB_1-94_-KaiC_DD_^1mer^ complex during incubation. These results suggest that Mg-ATP but not ATP reduced the affinity of KaiC^1mer^ for KaiB_1-94_ to dissociate the KaiB_1-94_-KaiC_DD_^1mer^ complex. When we examined the ATP- and Mg-ATP-induced oligomerization of KaiC_N_ in the KaiB_1-94_-KaiC_N_^1mer^ complex, we obtained essentially the same results; Mg-ATP, to a much greater extent than ATP, induced oligomerization of KaiC_N_ in the KaiB_1-94_-KaiC_N_^1mer^ complex (Figure 3B). These results suggest that Mg-ATP (and ATP) inhibited the association of KaiB_1-94_ with the N-terminal domain of KaiC.

### Effects of mutations in the ATPase motifs of KaiC on KaiB_1-94_-KaiC complex formation

**Figure 4:**
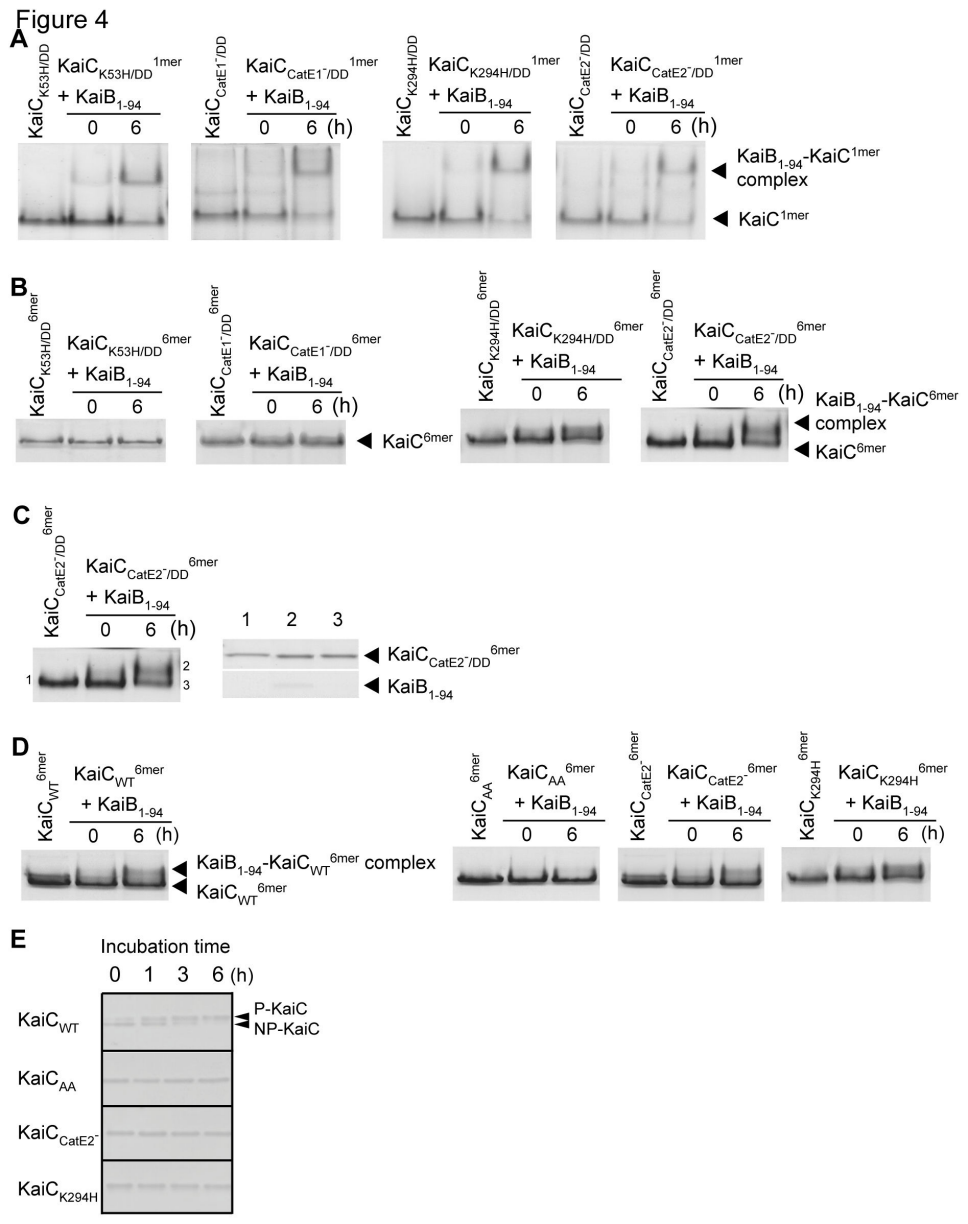
Effects of mutations in the ATPase motifs and phosphorylation sites of KaiC on formation of the KaiB_1-94_-KaiC^6mer^ complex. A. Native PAGE gels of the reaction products of KaiC^1mer^ with KaiB_1-94_. Reaction mixtures containing 15 μM KaiB_1-94_ and 5 μM KaiC^1mer^ were incubated at 4 °C for the periods indicated. Other conditions were the same as described for Figure 1. B. Native PAGE gels of the reaction products of KaiC^6mer^ with KaiB_1-94_. Reaction mixtures containing 5 μM KaiB_1-94_ and 1 μM KaiC^6mer^ were incubated in the presence of Mg-ATP at 4 °C for the periods indicated. Other conditions were the same as described for Figure 1C. C. A typical 2D SDS-PAGE gel from the native-PAGE gel shown in Figure 4B. The protein bands were excised, and the proteins were extracted from them and subjected to SDS-PAGE. Other conditions were the same as described for Figure 1C. The bands 1 to 3 were the KaiC_CatE2_-_/DD_^6mer^ band (control), the upper band of the reaction products of KaiC_CatE2_-_/DD_^6mer^ with KaiB_1-94_ incubated at 4 °C for 6 h, and the lower band of the reaction products of KaiC_CatE2_-_/DD_^6mer^ with KaiB_1-94_ incubated similarly. D. Native PAGE gels of the reaction products of KaiC^6mer^ with KaiB_1-94_. Reaction mixtures were incubated at 4 °C for the periods indicated. Other conditions were the same as described for Figure 4B expect that unphosphorylatable mutant KaiCs were used. E. SDS-PAGE gels showing no phosphorylation of KaiC_AA_^6mer^, KaiC_CatE2_-^6mer^, and KaiC_K294H_^6mer^. KaiCs^6mer^ (0.5 μM) were incubated with Mg-ATP in the presence of 0.5 μM KaiA at 40 °C for the periods indicated and then subjected to SDS-PAGE. p-KaiC, the phosphorylated forms of KaiC; np-KaiC, the unphosphorylated form of KaiC. Other conditions were the same as described for Figure 3.

 Both the N- and C-terminal domains of the KaiC subunit have a series of ATPase motifs (a Walker's motif A, a Walker's motif B, and a CatE [2]). When we examined the effects of mutations in those motifs on formation of KaiB_1-94_-KaiC^1mer^ complexes—using KaiCs with K53H and K294H mutations in Walker’s motif A and CatE1^-^ and CatE2^-^ mutations in CatEs [32]—we found that all the mutants we examined (KaiC_K53H/DD_, KaiC_CatE1_-_/DD_, KaiC_K294H/DD_, and KaiC_CatE2_-_/DD_) formed complexes with KaiB_1-94_ (Figure 4A and Table S1). Thus, none of the mutations in the ATPase motifs affected formation of the KaiB_1-94_-KaiC_DD_^1mer^ complex that occurs in the absence of ATP. 

When we examined KaiB_1-94_-KaiC^6mer^ complex formation in the presence of Mg-ATP, both KaiC_K294H/DD_^6mer^ and KaiC_CatE2_-_/DD_^6mer^, which have a C-terminal ATPase motif mutation, as well as KaiC_DD_ control, formed complexes with KaiB_1-94_ whereas KaiC_K53H/DD_^6mer^ and KaiC_CatE1_-_/DD_^6mer^, which have an N-terminal ATPase motif mutation, did not (Figure 4B). Native PAGE followed by SDS-PAGE revealed that all the candidate complex bands examined contained both KaiB_1-94_ and KaiC (a typical example is shown in Figure 4C). These results indicate that KaiC’s N-terminal ATPase motifs were responsible for formation of the KaiB_1-94_-KaiC^6mer^ complex that occurred in the presence of Mg-ATP. This observation is consistent with the finding described above that KaiC associates with KaiB_1-94_ *via* its N-terminal domain (Figure 2) and that Mg-ATP (and ATP) inhibits the association (Table S2). 

### Effects of the ATP, Mg-ATP, Mg-AMPPNP, and Mg-ATPγS used for the hexamerization of KaiC on KaiB_1-94_-KaiC^6mer^ complex formation

**Figure 5:**
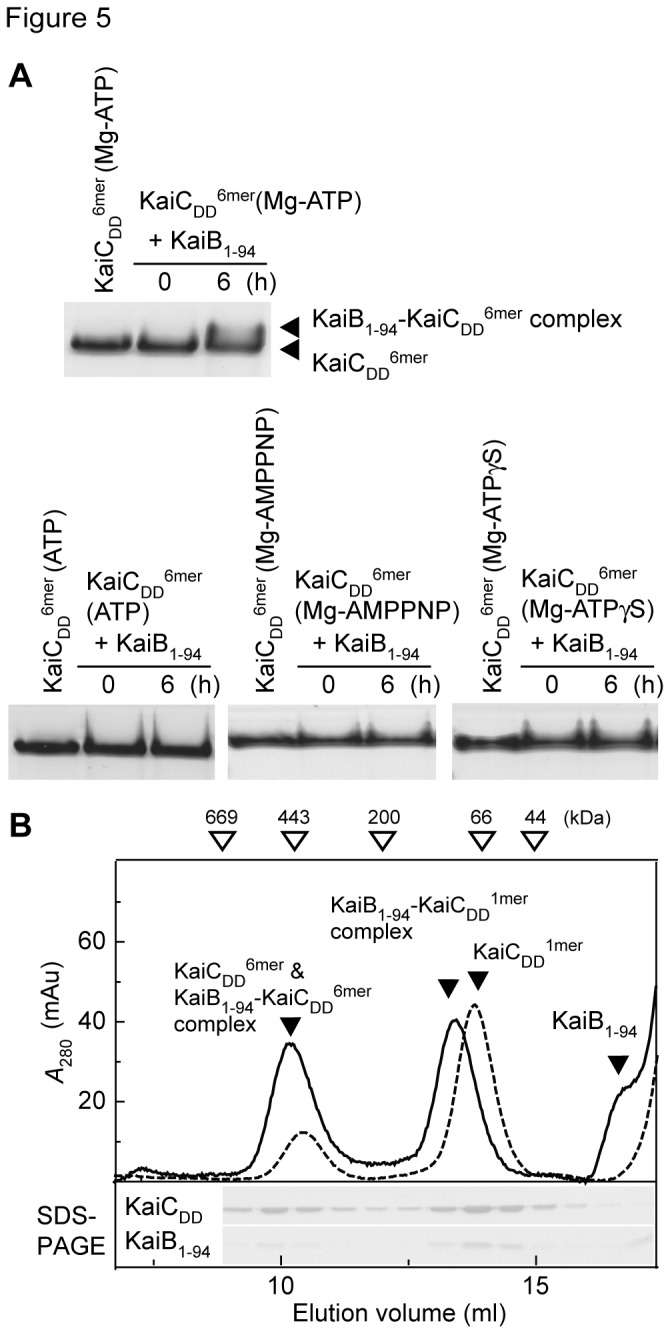
Effects of the ATP and ATP analogs used for the hexamerization of KaiC on formation of the KaiB_1-94_-KaiC_DD_^6mer^ complex assayed by native PAGE and gel filtration chromatography. A. Native PAGE gels. Reaction mixtures were incubated at 4 °C for the periods indicated. Other conditions were the same as described for Figure 4B except that KaiC_DD_^6mer^ (ATP), KaiC_DD_^6mer^ (Mg-AMPPNP), and KaiC_DD_^6mer^ (Mg-ATPγS) were used in the presence of 1 mM ATP, Mg-AMPPNP, and Mg-ATPγS, respectively. B. Gel filtration chromatography elution profiles of KaiC_DD_^6mer^ (Mg-ADP-AlF_X_) (dotted line) and a KaiB_1-94_-KaiC_DD_^6mer^ (Mg-ADP-AlF_X_) complex (solid line). KaiC_DD_^1mer^ (20 μM) was incubated in reaction buffer containing 6 mM ADP, 30 mM MgCl_2_, 2.5 mM NaF, and 2.5 mM AlCl_3_ at 25 °C for 2 h, and the reaction mixtures were mixed with an equal volume of 0 or 60 μM KaiB_1-94_ in the same buffer and then further incubated at 25 °C for 6 h. The reaction mixtures were then subjected to gel filtration chromatography on a Superdex 200/HR 10/30 column equilibrated with reaction buffer at 4 °C. Other conditions were the same as described for Figure 2.

 The observations described above suggest that ATP hydrolysis followed by the release of ADP from the N-terminal ATPase motifs are involved in KaiC^6mer^-KaiB_1-94_ complex formation. We therefore examined whether the KaiCs^6mer^ hexamerized by unhydrolyzable substrate analogs of ATPases, ATP (KaiC^6mer^ (ATP)), Mg-AMPPNP (KaiC^6mer^ (Mg-AMPPNP)), and Mg-ATPγS (KaiC^6mer^ (Mg-ATPγS)) formed a complex with KaiB_1-94_ and found that only KaiC^6mer^ hexamerized with Mg-ATP (KaiC^6mer^ (Mg-ATP)), which is a hydrolyzable substrate for ATPases, did (Figure 5A). These results support our hypothesis that hydrolysis of ATP in the N-terminal ATPase motifs is required for KaiB_1-94_-KaiC^6mer^ complex formation because ATP and the unhydrolyzable substrates analogs examined inhibited KaiB_1-94_-KaiC^6mer^ complex formation.

### KaiC^6mer^ (Mg-ADP-AlF_X_) forms a complex with KaiB_1-94_

 Because ADP-AlF_X_ mimics ADP-P_i_ [34], we used gel filtration chromatography to determine whether Mg-ADP-AlF_X_ hexamerizes KaiC_DD_^1mer^ and confirmed that it did (Figure 5B). Next, we examined the possible complexes formed by KaiC_DD_^6mer^ (Mg-ADP-AlFx) with KaiB_1-94_. KaiC_DD_^6mer^ (Mg-ADP-AlF_X_) formed a complex with KaiB_1-94_ (Figure 5B), but we also detected a KaiB_1-94_-KaiC_DD_^1mer^ complex under conditions wherein a substantial amount of KaiC_DD_^1mer^ was not hexamerized (Figure 5B). This observation supports our above conclusion that ATP hydrolysis is required for formation of the KaiB_1-94_-KaiC_DD_^6mer^ complex. Therefore, the conformation of KaiC_DD_^6mer^ (Mg-ADP-P_i_) that allows complex formation with KaiB_1-94_ may differ from that of KaiC_DD_^6mer^ (Mg-ATP) without ATP hydrolysis and that of KaiC^6mer^ (ATP). 

### Effects of KaiC phosphorylation-site mutations and autophosphorylation mutations on KaiB_1-94_-KaiC^6mer^ complex formation

 We examined the effects of mutations in the two phosphorylation sites of KaiC on KaiB_1-94_-KaiC^6mer^ complex formation. While KaiC_WT_^6mer^ (Figure 4D) and KiaC_DD_^6mer^ (Figure 5A) formed a complex with KaiB_1-94_, KaiC_AA_^6mer^ did not form such a complex (Figure 4D). These results are consistent with previous reports showing that phosphorylated KaiC (KaiC_DD_ and KaiC_DE_, a mutant KaiC similar to KaiC_DD_) but not unphosphorylated KaiC (KaiC_AA_) formed a complex with KaiB_WT_ [5,16-18]. When KaiC_WT_^6mer^ was incubated with Mg-ATP in the presence of KaiA, it was highly phosphorylated (Figure 4E) [8,9,13,21,27,35]. The two upper bands correspond to the phosphorylated forms of KaiC whereas the lowest band corresponds to the unphosphorylated form of KaiC (Figure 4E) [3,13]. KaiC_AA_^6mer^, KaiC_CatE2_-^6mer^, and KaiC_K294H_^6mer^ did not show any phosphorylated bands even in the presence of KaiA (Figure 4E). 

 Next, we examined the effects of KaiC autophosphorylation mutations on KaiB_1-94_-KaiC^6mer^ complex formation. In spite of lacking the autophosphorylation [13], both KaiC_K294H_^6mer^ and KaiC_CatE2_-^6mer^ formed complexes with KaiB_1-94_ (Figure 4D). Thus, phosphorylation of KaiC *per se* was not essential for KaiB_1-94_-KaiC^6mer^ complex formation, although KaiC’s phosphorylation state might indirectly modulate complex formation.

### Effects of KaiC phosphorylation-site mutations on the *N-*terminal ATPase activity of KaiC

**Figure 6:**
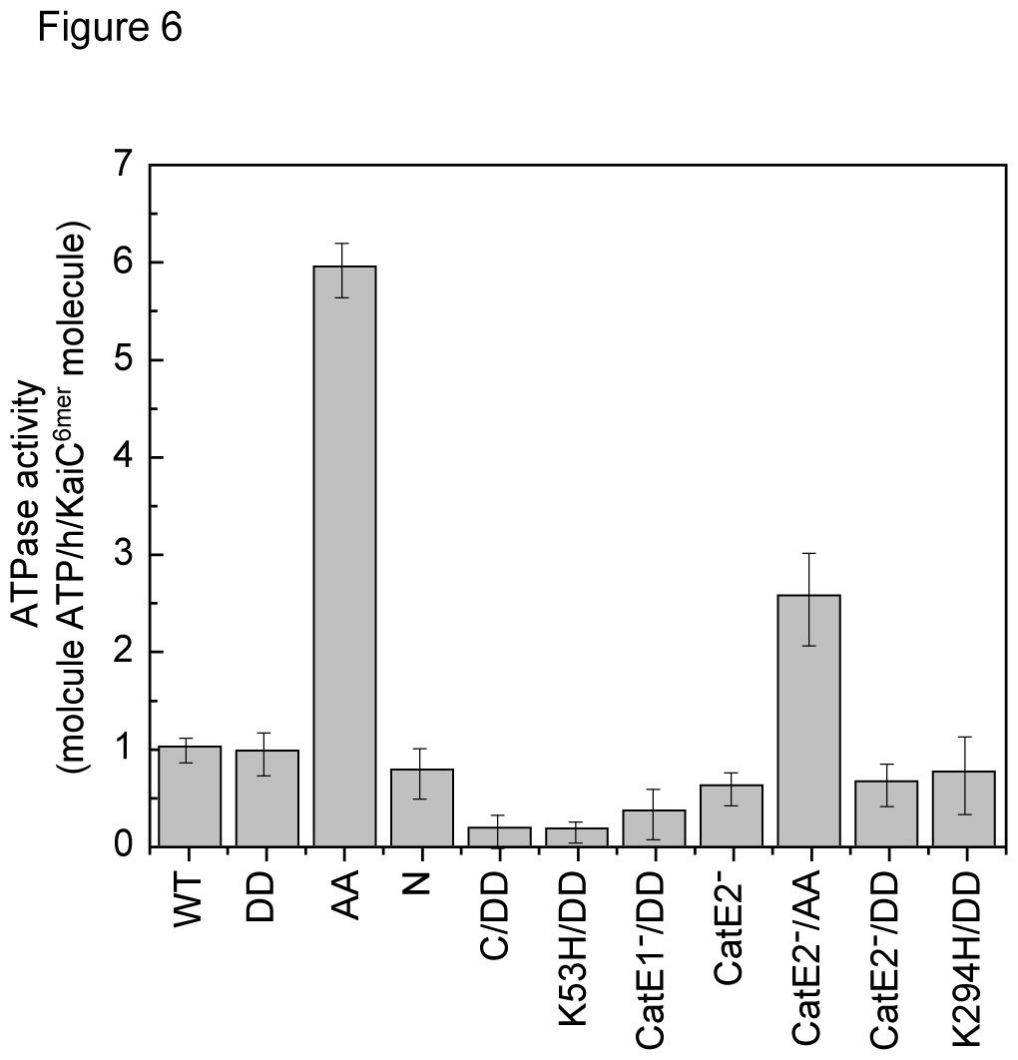
Effects of mutations in the ATPase motifs and phosphorylation sites of KaiC on KaiC’s ATPase activity. We incubated 1 μM KaiCs^6mer^ with Mg-ATP in reaction buffer at 4 °C for 6 h and then measured ATPase activities. Values are means ± SD from triplicate assay. KaiCs^6mer^: WT, KaiC_WT_^6mer^; DD, KaiC_DD_^6mer^; AA, KaiC_AA_^6mer^; N, KaiC_N_^6mer^; C/DD, KaiC_C/DD_^6mer^; K53H/DD, KaiC_K53H/DD_^6mer^; CatE1^-^/DD, KaiC_CatE1_-_/DD_^6mer^; CatE2^-^, KaiC_CatE2_-^6mer^; CatE2^-^/AA, KaiC_CatE2_-_/AA_^6mer^; CatE2^-^/DD, KaiC_CatE2_-_/DD_^6mer^; K294H/DD, KaiC_K294H/DD_^6mer^.

 To examine the effects of mutations in the two phosphorylation sites of KaiC on the N-terminal ATPase activity of KaiC, we compared the ATPase activities of KaiC_CatE2_-_/AA_^6mer^, KaiC_CatE2_-_/DD_^6mer^, and KaiC_CatE2_-^6mer^ because KaiC_CatE2_- lacks the C-terminal ATPase activity of KaiC [13]. The ATPase activities of KaiC_CatE2_-_/DD_^6mer^ and KaiC_CatE2_-^6mer^ reflect the N-terminal ATPase activity while that of KaiC_DD_^6mer^ and KaiC_WT_^6mer^ reflect the total ATPase activity. The former activities were approximately half of the latter activities (Figure 6 and Table S4). These four KaiCs^6mer^ formed a complex with KaiB_1-94_ (Tables S2 and S3). On the other hand, the ATPase activity of KaiC_AA_^6mer^, which did not form a complex with KaiB_1-94_ (Table S3), showed 6 times higher ATPase activity than KaiC_DD_^6mer^ and KaiC_WT_^6mer^ (Figure 6). In consistent with this, KaiC_AA_ has been reported to show 2.5 times higher ATPase activity than KaiC_DE_, which is a mutant KaiC similar to KaiC_DD_ [4]. The ATPase activity of KaiC_CatE2_-_/AA_, which formed a complex with KaiB_1-94_ that could only be detected by silver staining (Table S3), was more than 3 times as high as those of KaiC_CatE2_-_/DD_^6mer^ and KaiC_CatE2_-^6mer^. The ATPase activities of KaiC_CatE2_-_/AA_^6mer^, KaiC_CatE2_-_/DD_^6mer^, and KaiC_CatE2_-^6mer^ all reflect the N-terminal ATPase activity of KaiC. Thus, although the N-terminal ATPase activity of KaiC is probably required for KaiC^6mer^ to form a complex with KaiB_1-94_, its excessively high activity (KaiC_AA_^6mer^ and KaiC_CatE2_-_/AA_^6mer^) may inhibit complex formation. The ATPase activity of KaiC_CatE1_-_/DD_^6mer^ reflecting the C-terminal ATPase activity of KaiC was almost the same as that of KaiC_C/DD_^6mer^ and approximately half of that of KaiC_DD_^6mer^ (Figure 6 and Table S4), as described previously [10]. 

## Discussion

 Since KaiC^1mer^ formed a complex with KaiB_1-94_ (Figure 1C), the KaiC subunit *per se* is able to form a complex with KaiB. Recently, NMR analysis revealed that KaiC^1mer^ forms a complex with KaiB *via* its N-terminal domain [29], and here KaiC (KaiC^6mer^ as well as KaiC^1mer^) formed a complex with KaiB_1-94_ *via* its N-terminal domain (Figure 2). ATP, Mg-ATP, Mg-AMPPNP, and Mg-ATPγS hexamerize KaiC, probably by binding subunits [13,32,36,37]. KaiC^6mer^ (ATP), KaiC^6mer^ (Mg-AMPPNP), and KaiC^6mer^ (Mg-ATPγS) did not form a complex with KaiB_1-94_ (Figure 5A). This indicates that when KaiC-bound ATP (or ATP analogs) is unhydrolyzable, KaiC does not form a complex with KaiB. KaiC_DD_^6mer^ (Mg-ADP-AlF_X_), which mimics the Mg-ADP-Pi state of KaiC_DD_^6mer^, formed a complex with KaiB_1-94_ (Figure 5B). It is likely, therefore, that ATP regulates KaiB-KaiC^6mer^ complex formation by hindering complex formation by KaiC^6mer^-bound ATP, and its hydrolysis is required for complex formation. 

That mutations in the N-terminal but not its C-terminal ATPase motifs affected the complex formation of KaiC^6mer^ (Mg-ATP) with KaiB_1-94_ (Figure 4B) indicates that ATP hydrolysis by the N-terminal KaiC’s ATPase motifs is responsible for KaiB-KaiC^6mer^ complex formation. These results are consistent with a recent report [38]. Because the KaiC_N_^6mer^-KaiB_1-94_ complex rapidly dissociated into a KaiB_1-94_-KaiC_N_^1mer^ complex (Figure 2C), the partial dissociation (or relaxation) of the N-terminal domains of KaiC^6mer^ probably occurred on the interaction of KaiC^6mer^ with KaiB. This partial dissociation of the KaiC^6mer^ N-terminal domains is likely required for formation of KaiB-KaiC^6mer^ complex *via* KaiC’s N-terminal domain. The relaxation of the KaiC^6mer^ N-terminal domains on interaction of KaiB has been revealed recently by NMR analysis [29].

The KaiB molecule, which is a homotetramer organized as a dimer of dimers (KaiB^4mer^) [24], probably dissociates into two dimers (KaiB^2mer^) on interaction with KaiC^6mer^ and forms a complex comprising one molecule of KaiC^6mer^ and two molecules of KaiB^2mer^ [17], as suggested by cryo-electron microscopy analysis [25]. We have proposed that the positively charged cleft (PC) of the KaiB^4mer^ molecule, where the functionally important KaiB residues are concentrated, is an active site(s) required for interaction with KaiA and KaiC [24,28,39]. The PC of KaiB^4mer^, which is located on the dimer-dimer interface [24], is probably exposed by dissociation of KaiB^4mer^ into dimers to interact with KaiC^6mer^ [28,39], whereas the corresponding region of KaiB_1-94_, a dimeric mutant of KaiB, is always exposed [17]. Two areas on KaiC^6mer^ molecule, on the other hand, are highly negatively charged—one around and inside the pore of KaiC^6mer^ N-terminal domains and the other around the inter-subunit interface of one of two adjacent KaiC^6mer^ N-terminal domains (Figures S1A and S1B) [37]. Interestingly, the ATP bound to the N-terminal ATPase motifs (namely, ATP-binding sites) is located adjacent to the latter area of KaiC (Figure S1B) [37]. Electrostatic interaction between the PC on KaiB and the aforementioned area of KaiC may allow sequestration of KaiB^4mer^ (also KaiB^2mer^ such as KaiB_1-94_) and induce dissociation into dimers (temporal weak association). Then, the dissociation of two adjacent N-terminal domains in KaiC^6mer^ resulting from the hydrolysis of ATP bound to the N-terminal ATPase motifs on one of the two adjacent subunits (Figures S1C and S1D), which pastes the two N-terminal domains each other [13,32,36,37], may expose the latter area of KaiC—a possible KaiB-interacting surface—to KaiB^2mer^, and electrostatic interaction between the PC on KaiB and the latter area of KaiC may result in the tight association of KaiB^2mer^ with KaiC^6mer^. The ATP bound to the N-terminal ATPase motifs inhibits the association of KaiB^2mer^ with KaiC^6mer^ *via* KaiC N-terminal domains, as demonstrated in KaiB_1-94_-KaiC^6mer^ complex formation (Figure 3C). Thus, we calculated the surface potentials of KaiC^6mer^ without ATP (Figure S1C) and with ATP (Figure S1D) and found them to be almost the same and unlikely to affect the interaction of KaiC with KaiB. While KaiB_WT_^4mer^ formed a complex formation with KaiC^6mer^ (Figure 1B), it did so only slightly with KaiC^1mer^ (Figure 1D). KaiB_1-94_, in contrast, formed a complex with both KaiC^6mer^ and KaiC^1mer^ (Figures 1B, 1C and 5A). Therefore, interaction with KaiC^6mer^ but not with KaiC^1mer^ likely enhanced KaiB^4mer^ dimerization, suggesting the possibility that enhancement requires the hexameric structure of KaiC^6mer^ N-terminal domains. 

 It has been previously proposed that the phosphorylation state of KaiC was involved in its forming a complex with KaiB [18]. However, our data described here showing that KaiC_K294H_^6mer^ and KaiC_CatE2_-^6mer^, which lack the autokinase activity (Figure 4E) [13], formed complexes with KaiB_1-94_ (Figure 4D) indicated that the phosphorylated state of KaiC is not essential for KaiB-KaiC^6mer^ complex formation. Our results are consistent with the recently reported results that KaiC_CatE2_-^6mer^ formed a complex with KaiB_WT_ [38]. The phosphorylation state of KaiC, therefore, is not directly involved in and essential for complex formation. 

**Figure 7:**
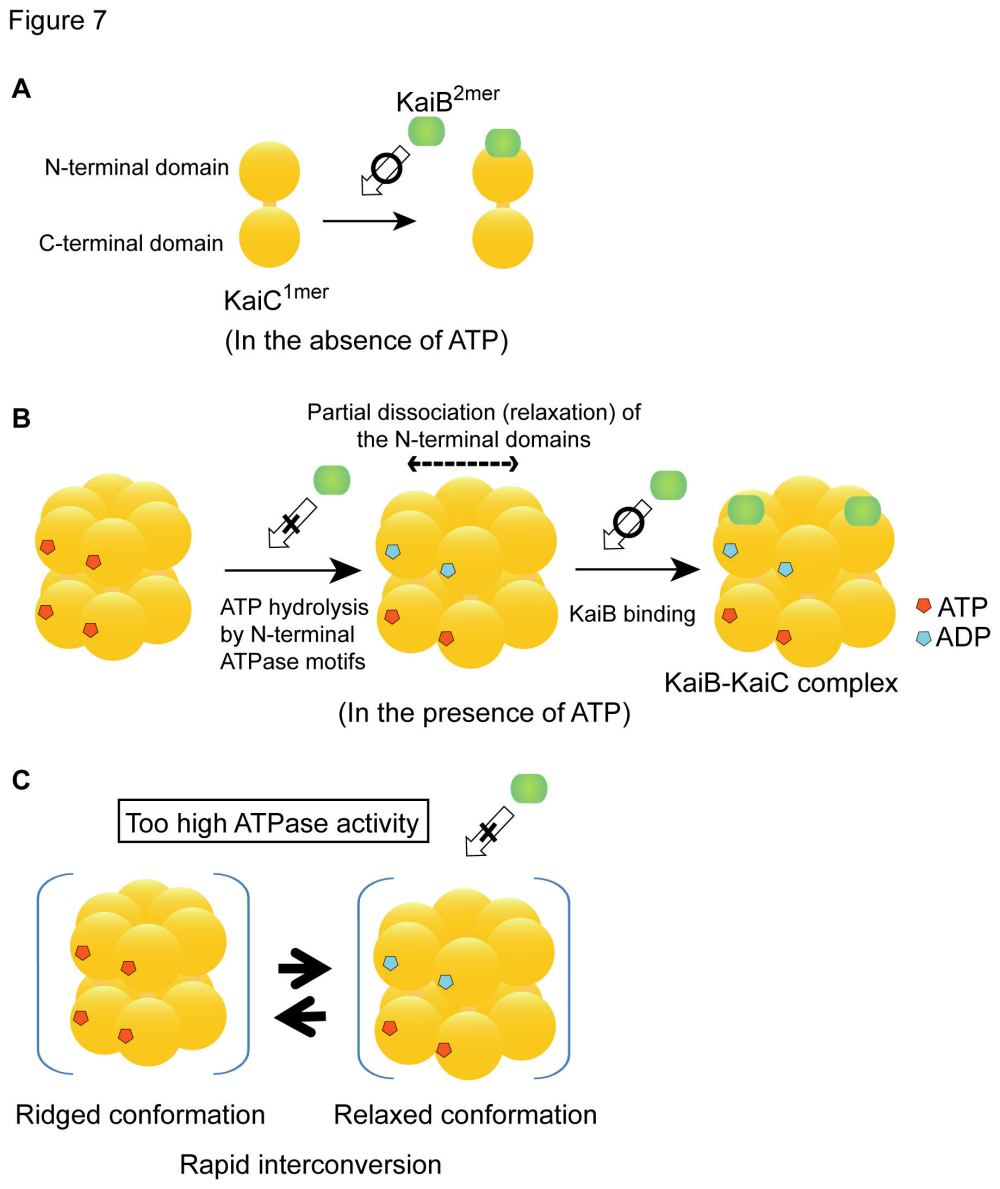
ATP-mediated regulation model for KaiB-KaiC interaction. A. Interaction between KaiB and KaiC^1mer^. B. Partial dissociation (relaxation) of the N-terminal domain of KaiC^6mer^ and complex formation of one KaiC^6mer^ molecule with 2 KaiB^2mer^ molecules. For simplification, we express Mg-chelated ATP and ADP as ATP and ADP. The nucleotide state of the C-terminal ATP-binding site (ATPase motifs) of KaiC^6mer^ is not known. C. Rapid interconversion between the rigid ATP-bound and relaxed ATP-hydrolyzed form (ADP-bound or unbound) conformations in the N-terminal domains of KaiC^6mer^.

 KaiC_AA_^6mer^ and KaiC_CatE2_-_/AA_^6mer^, which did not form a complex with KaiB_1-94_, showed much higher ATPase activity than any other KaiC ATPase motif mutants we examined (Figure 6 and Table S4). The excessively high ATPase activity of the N-terminal ATPase motifs of KaiC_AA_^6mer^, which bounces in and out of the ATP bound to the N-terminal ATPase motifs, may hinder formation of the KaiB-KaiC^6mer^ complex. In KaiC^6mer^, the phosphorylation state of the C-terminal domain could affect its association with KaiB *via* the N-terminal domain through modulating the N-terminal ATPase activity. However, we cannot exclude a possibility that KaiC_AA_^6mer^, which is likely not a perfect mimic for the fully unphosphorylated form of KaiC, might have a changed structure, which might enhance its ATPase activity but might reduce its association with KaiB. We propose the following model for ATP regulation of KaiB-KaiC interaction. The KaiC subunit is able to form a complex with KaiB (Figure 7A). The N-terminal domains of KaiC^6mer^ are partially dissociated (relaxed) when the ATP bound to the N-terminal ATPase motifs that pastes adjacent N-terminal domains each other in KaiC^6mer^ is hydrolyzed (Figure 7B), which allows KaiB to associate with the KaiC^6mer^ N-terminal domains. Then, KaiC^6mer^-associated KaiB suppresses the ATPase activity of KaiC^6mer^ [4] by inhibiting ATP binding to KaiC^6mer^ N-terminal domains. KaiC_AA_^6mer^ and KaiC_CatE2_-_/AA_^6mer^, which seem to mimic the unphosphorylation state of KaiC, have excessively high N-terminal ATPase activity (Figure 6 and Table S4), and that may cause rapid interconversion of the ridged (ATP-bound) and relaxed (ATP-hydrolyzed; ADP-bound or unbound) conformations of the N-terminal domains in KaiC^6mer^. We propose here that this rapid interconversion inhibits KaiB-KaiC^6mer^ complex formation though we cannot explain this inhibiting mechanism at present (Figure 7C). 

 The C-terminal ATPase motifs of KaiC are involved in the hexamerization of KaiC C-terminal domains [13] as well as their inter-subunit autophosphorylation [8] and probably autodephosphorylation [12]. Although the KaiC N-terminal ATPase motifs are involved in the hexamerization of KaiC N-terminal domains [13], and the affinity of the N-terminal ATPase motifs for ATP is higher than that of its C-terminal ATPase motifs [13], and therefore, the N-terminal domains are likely to be more tightly connected than the C-terminal domains [13,16,29], the function of the N-terminal ATPase motifs remains unknown. In this investigation, we have succeeded in revealing that the nucleotide state of the N-terminal ATPase motifs regulates KaiB-KaiC interaction. Because KaiB and SasA competitively associate with KaiC *via* KaiC N-terminal domains [14,17], the nucleotide state also can regulate KaiC-SasA interaction *via* the KaiB-KaiC interaction. ATP acts not only as a biological fuel, but also as a physiological regulator. There are some examples for ATP regulation of the physiological function. Many different cell types release ATP in response to mechanical or biochemical stimulation, and the released ATP modulates cell function by activating nearby purinoceptors, such as ion channel P2X receptors and G-protein-coupled P2Y receptors [40-42].

## Supporting Information

Figure S1 **Electrostatic surface potential of KaiC^6mer^ and KaiC^1mer^ of *Synechococcus* KaiC (PDB code: 2GBL).** We calculated electrostatic surface representations of KaiC^6mer^ (A, B) and KaiC^1mer^ (C, D) using the PyMOL plug-in APBS [43]. A. Top view of the N-terminal domain of KaiC^6mer^ with ATP. B. Side view of KaiC^6mer^ with ATP. Interface of KaiC without ATP (C) and with ATP (D). The saturation thresholds were -5 and +5. For electrostatic surface potential: blue, positive; red, negative. Arrows indicated the negatively charged areas.
(TIF)

Table S1 **Complex formation of KaiCs^1mer^ with KaiB_1-94_.**
(DOC)

Table S2 **Formation of KaiB_1-94_-KaiCs^6mer^ complex.**
(DOC)

Table S3 **Complex formation of unphosphorylatable KaiCs^6mer^ with KaiB_1-94_.**
(DOC)

Table S4 **Effects of mutations in the ATPase motifs and phosphorylation sites on KaiC ATPase activity.**
(DOC)
