# Supplementary figures and images for: The ATP-Mediated Regulation of KaiB-KaiC Interaction in the Cyanobacterial Circadian Clock
Source: PLoS One. 2013 Nov 11;8(11):e80200. doi: 10.1371/journal.pone.0080200 (PMC3823767; doi:10.1371/journal.pone.0080200)

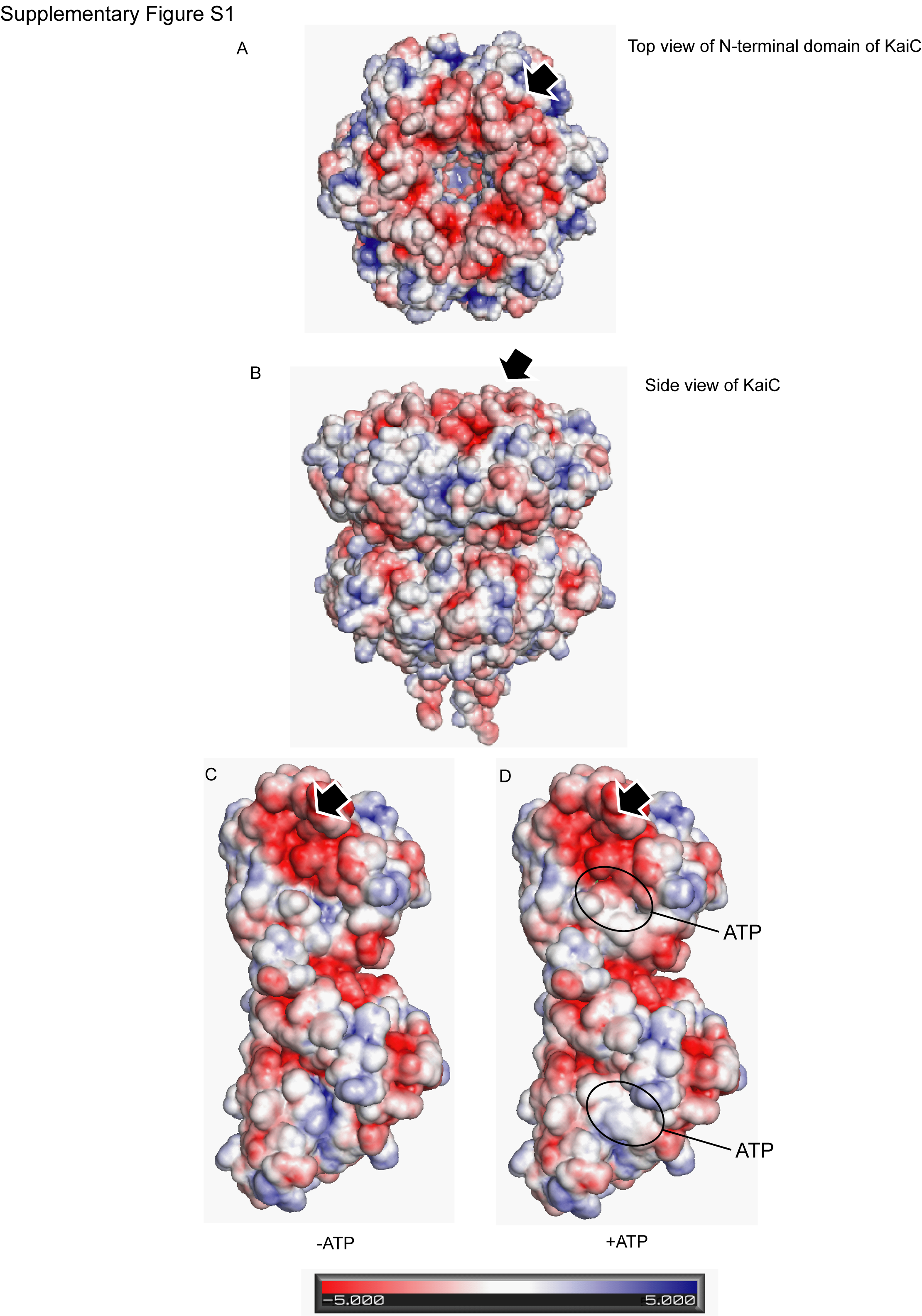

Supplement: Figure S1 — Electrostatic surface potential of KaiC6mer and KaiC1mer of Synechococcus KaiC (PDB code: 2GBL). We calculated electrostatic surface representations of KaiC6mer (A, B) and KaiC1mer (C, D) using the PyMOL plug-in APBS [43]. A. Top view of the N-terminal domain of KaiC6mer with ATP. B. Side view of KaiC6mer with ATP. Interface of KaiC without ATP (C) and with ATP (D). The saturation thresholds were -5 and +5. For electrostatic surface potential: blue, positive; red, negative. Arrows indicated the negatively charged areas. (TIF) [file pone.0080200.s001.tif]
