# Supplementary material for: The ATP-Mediated Regulation of KaiB-KaiC Interaction in the Cyanobacterial Circadian Clock
Source: PLoS One. 2013 Nov 11;8(11):e80200. doi: 10.1371/journal.pone.0080200 (PMC3823767; doi:10.1371/journal.pone.0080200)
Supplement: Table S1 — Complex formation of KaiCs1mer with KaiB1-94. (DOC) [file pone.0080200.s002.doc]

Table S1. Complex formation of KaiCs^1mer^ with KaiB_1-94_.

| KaiC^1mer^ | Native PAGE | Gel filtration chromatography |
| --- | --- | --- |
| KaiC_N_ | +^*^ | + |
| KaiC_C/DD_ | -^†^ | - |
| KaiC_WT_ (control) | + |  |
| KaiC_DD_ | + |  |
| KaiC_CatE1_-_/DD_ | + |  |
| KaiC_K53H/DD_ | + |  |
| KaiC_CatE2_-_/DD_ | + |  |
| KaiC_K294H/DD_ | + |  |

^*^formation of KaiB_1-94_-KaiC^1mer^ complex

^†^no detectable complex formation

Reaction mixtures containing 15 μM KaiB_1-94_ and 5 μM KaiCs^1mer^ in reaction buffer and those containing 36 μM KaiB_1-94_ and 12 μM KaiCs^1mer^ in the buffer were incubated at 4 °C for 6 h, and then subjected to native PAGE and gel filtration chromatography. Other native-PAGE conditions were the same as described for Figure 1A, and those for gel filtration chromatography were the same as described for Figure 2C except that reaction and gel filtration chromatography buffers did not contain ATP and MgCl_2_.
