# Supplementary material for: The ATP-Mediated Regulation of KaiB-KaiC Interaction in the Cyanobacterial Circadian Clock
Source: PLoS One. 2013 Nov 11;8(11):e80200. doi: 10.1371/journal.pone.0080200 (PMC3823767; doi:10.1371/journal.pone.0080200)
Supplement: Table S2 — Formation of KaiB1-94-KaiCs6mer complex. (DOC) [file pone.0080200.s003.doc]

Table S2. Formation of KaiB_1-94_-KaiCs^6mer^ complex

| KaiC^6mer^ | Native-PAGE | | Gel filtration chromatography |
| --- | --- | --- | --- |
|  | KaiC^6mer^ (Mg-ATP) | KaiC^6mer^ (ATP) | KaiC^6mer^ (Mg-ATP) |
| KaiC_N_ | - | - | + |
| KaiC_C/DD_ | - | - | - |
| KaiC_DD_ | + | - |  |
| KaiC _CatE1_-_/DD_ | - |  | ±^*^ |
| KaiC_K53H/DD_ | - |  | ±^*^ |
| KaiC_CatE2_-_/DD_ | + | - |  |
| KaiC_K294H/DD_ | + | - |  |

^*^ KaiC_CatE1_-_/DD_^6mer^ and KaiC_K53H/DD_^6mer^ formed a complex with KaiB_1-94_ that was detected only by immunoblot analysis

For native PAGE, reaction mixtures containing 5 μM KaiB_1-94_, 1 μM KaiCs^6mer^, and 1 mM ATP and those containing 5 μM KaiB_1-94_, 1 μM KaiCs^6mer^, and Mg-ATP were incubated at 4 °C for 6 h. For gel filtration chromatography, reaction mixtures containing 15 μM KaiB_1-94_, 2.5 μM KaiCs^6mer^, and Mg-ATP were incubated similarly. Other conditions were the same as described in the Table S1 footnote.
