# Supplementary material for: The ATP-Mediated Regulation of KaiB-KaiC Interaction in the Cyanobacterial Circadian Clock
Source: PLoS One. 2013 Nov 11;8(11):e80200. doi: 10.1371/journal.pone.0080200 (PMC3823767; doi:10.1371/journal.pone.0080200)
Supplement: Table S3 — Complex formation of unphosphorylatable KaiCs6mer with KaiB1-94. (DOC) [file pone.0080200.s004.doc]

Table S3. Complex formation of unphosphorylatable KaiCs^6mer^ with KaiB_1-94_.

| KaiC^6mer^ | Native-PAGE | Gel filtration chromatography |
| --- | --- | --- |
| KaiC_WT_^6mer^ （control) | + |  |
| KaiC_DD_^6mer^ （control) | + |  |
| KaiC_AA_^6mer^ | - | ±^†^ |
| KaiC_CatE2_-^6mer^ | + |  |
| KaiC_K294H_^6mer^ | + |  |
| KaiC_CatE2_-_/AA_^6mer^ | ±^*^ | ±^†^ |

^*^ KaiC_CatE2_-_/AA_^6mer^ formed a complex with KaiB_1-94_ that was detected only by silver staining.

^†^ KaiC_AA_^6mer^ and KaiC_CatE2_-_/AA_^6mer^ formed a complex with KaiB_1-94_ that was detected only by immunoblot analysis

Unphosphorylatable KaiCs^6mer^ (Mg-ATP) were used. Other conditions were the same as described in the Table S2 footnote except that reaction mixtures contained Mg-ATP.
