# Supplementary material for: The ATP-Mediated Regulation of KaiB-KaiC Interaction in the Cyanobacterial Circadian Clock
Source: PLoS One. 2013 Nov 11;8(11):e80200. doi: 10.1371/journal.pone.0080200 (PMC3823767; doi:10.1371/journal.pone.0080200)
Supplement: Table S4 — Effects of mutations in the ATPase motifs and phosphorylation sites on KaiC ATPase activity. (DOC) [file pone.0080200.s005.doc]

Table S4. Effects of mutations in the ATPase motifs and phosphorylation sites on KaiC ATPase activity.

|  | KaiC^6mer^ | ATPase activity  (molecule ATP/h/molecule) | | |
| --- | --- | --- | --- | --- |
|  | KaiC_WT_^6mer^ |  | 1.0 | ± 0.13 |
|  | KaiC_DD_^6mer^ |  | 0.99 | ± 0.22 |
|  | KaiC_AA_^6mer^ |  | 6.0 | ± 0.28 |
|  | KaiC_N_^6mer^ |  | 0.78 | ± 0.26 |
|  | KaiC_C/DD_^6mer^ |  | 0.18 | ± 0.17 |
|  | KaiC_K53H/DD_^6mer^ |  | 0.17 | ± 0.11 |
|  | KaiC_CatE1_-_/DD_^6mer^ |  | 0.36 | ± 0.26 |
|  | KaiC_CatE2_-^6mer^ |  | 0.62 | ± 0.17 |
|  | KaiC_CatE2_-_/AA_^6mer^ |  | 2.6 | ± 0.48 |
|  | KaiC_CatE2_-_/DD_^6mer^ |  | 0.66 | ± 0.22 |
|  | KaiC_K294H_^6mer^ |  | 0.76 | ± 0.40 |

Data shown are from the experiments described in Figure 6.
